# Two-Dimensional DOA Estimation for Coprime Planar Arrays: From Array Structure Design to Dimensionality-Reduction Root MUSIC Algorithm

**DOI:** 10.3390/s25051456

**Published:** 2025-02-27

**Authors:** Yunhe Shi, Xiaofei Zhang, Shengxinlai Han

**Affiliations:** College of Electronic and Information Engineering, Nanjing University of Aeronautics and Astronautics, Nanjing 211106, China; zhangxiaofei@nuaa.edu.cn (X.Z.); hanshengxinlai@nuaa.edu.cn (S.H.)

**Keywords:** sparse array design, 2-D DOA estimation, coprime planar array, array signal processing, degrees of freedom (DOFs)

## Abstract

This paper proposes a novel sparse array design and an efficient algorithm for two-dimensional direction-of-arrival (2D-DOA) estimation. By analyzing the hole distribution in coprime arrays and introducing supplementary elements, we design a Complementary Coprime Planar Array (CCPA) that strategically fills key holes in the virtual array. This design enhances the array’s continuous Degrees Of Freedom (DOFs) and virtual aperture, achieving improved performance in 2D-DOA estimation with fewer physical elements. The virtualization of the array further increases the available DOFs, while the hole-filling strategy ensures better spatial coverage and continuity. On the algorithmic side, we introduce a dimensionality-reduction root MUSIC algorithm tailored for uniform planar arrays after virtualization. By decomposing the two-dimensional spectral peak search into two one-dimensional polynomial root-finding problems, the proposed method significantly reduces computational complexity while maintaining high estimation accuracy. This approach effectively mitigates the challenges of 2D peak search, making it computationally efficient without sacrificing precision. Extensive simulations demonstrate the advantages of the proposed array and algorithm, including higher DOFs, reduced complexity, and superior estimation performance compared to existing methods. These results validate the effectiveness of the proposed framework in advancing sparse array design and signal processing for 2D-DOA estimation.

## 1. Introduction

Sensor array signal processing is a crucial field within modern signal processing, involving the arrangement of multiple sensors at different spatial positions to form a coherent array. This configuration enables the reception and analysis of spatial signals by sampling distributed field data and converting them into discrete spatial observations. The primary objectives are to enhance desired signals, suppress noise and interference, and extract valuable information for parameter estimation [1,2,3,4,5,6]. By processing the data acquired by the array, this approach improves the accuracy and reliability of signal detection across diverse applications. It has found widespread use in research and engineering domains, including wireless communication, radar systems, underwater acoustics, medical imaging, and speech signal processing [7,8,9,10,11,12,13,14,15,16]. Research in this area typically focuses on developing advanced techniques to optimize signal acquisition, enhance processing efficiency, and tackle challenges posed by complex environments. A representative research area within sensor array signal processing is spatial spectrum estimation, commonly referred to as direction-of-arrival (DOA) estimation. This technique utilizes antenna arrays to capture target signals in noisy and interference-prone environments and subsequently derives the directions of the signal sources through specialized processing methods. Several approaches exist for DOA estimation, such as beamforming, which focuses the array’s main lobe in the desired direction while suppressing interference [17]. Subspace-based methods, including the MUSIC and ESPRIT algorithms, offer super-resolution capabilities, surpassing the classical Rayleigh limit [18]. Building on one-dimensional DOA methods, researchers have developed two-dimensional techniques, such as 2D-MUSIC [19], 2D-ESPRIT [20], and the DOA matrix method [21], enabling the simultaneous estimation of azimuth and elevation angles. While these methods, often relying on uniform planar arrays, achieve high accuracy, they demand a large number of array elements, increasing system complexity and computational costs. To mitigate these challenges, various sparse array structures have been proposed, including coprime arrays [22,23], nested arrays [24], Minimum Redundancy Linear Arrays (MRLAs) [25,26,27], and Minimum Hole Arrays (MHAs) [27,28]. Unlike traditional dense uniform arrays, sparse arrays feature wider element spacings, which expand the effective aperture while reducing mutual coupling and noise coherence. Sparse arrays further exploit high-order statistics of received signals to construct equivalent virtual arrays, such as the sum or difference co-array (SDCA), enabling underdetermined DOA estimation. This allows for detection of more targets than the physical number of sensors, significantly reduced hardware costs. However, certain sparse array designs, like MRLAs and MHAs, rely on exhaustive searches for optimal element positioning, limiting their practicality. Coprime planar arrays, created by combining two sparse uniform arrays with large spacings, alleviate mutual coupling effects but introduce gaps (or “holes”) in the difference co-array (DCA), which diminish the degrees of freedom (DOFs). To address this limitation, researchers have proposed “hole-filling” strategies, where additional elements are strategically placed to create a continuous DCA. This approach enhances the DOFs, thereby improving the array’s performance while maintaining cost efficiency. To further tackle computational challenges, this paper presents a MUSIC algorithm based on dimensionality reduction and root finding. By reformulating the two-dimensional spectral peak search into two one-dimensional estimation problems and, subsequently, into a polynomial root-solving task, the proposed method significantly reduces the computational complexity of traditional two-dimensional MUSIC algorithms.

The main contributions of this work are summarized as follows:

Firstly, to address the issue of reduced degrees of freedom (DOFs) caused by holes in the virtualized traditional coprime array, we propose the CCPA (coprime planar array) design with hole filling. By leveraging a hole-filling strategy, this method increases the number of continuous virtual array elements and expands the virtual array aperture. Compared to conventional approaches, the CCPA achieves a substantial improvement in DOFs. Secondly, to mitigate the high computational complexity of dimensionality-reduced MUSIC algorithms, we introduce the dimensionality-reduced root MUSIC algorithm. This approach significantly lowers computational demands while maintaining high estimation accuracy. Finally, the effectiveness of the proposed array structure and algorithm is demonstrated through simulations, showing clear advantages over traditional arrays and algorithms.

The remainder of this paper is organized as follows. Section 2 examines the hole patterns in coprime planar arrays (CPAs) and introduces the proposed sparse array design based on hole filling. In Section 3, we present the new MUSIC-based two-dimensional DOA estimation algorithm. Section 4 offers simulations and performance analysis, and conclusions are drawn in Section 5.

## 2. Sparse Array Design Based on Hole Filling

This section analyzes the limitations of traditional coprime planar arrays (CPAs) and proposes the complementary coprime planar array (CCPA) design to enhance the degrees of freedom and virtual aperture.

### 2.1. Coprime Planar Arrays

**Figure 1:**
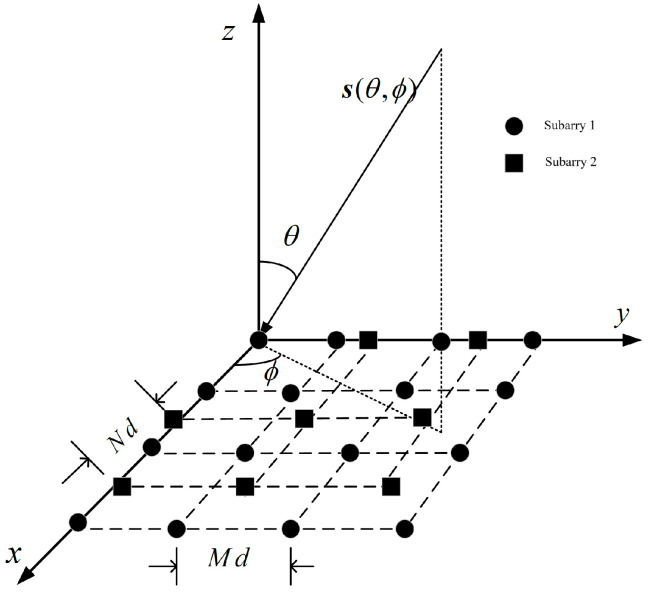
Schematic diagram of coprime array structure.

The coprime array consists of two uniform subarrays. Subarray 1 is an N×N square matrix with element spacing of Md, and subarray 2 is a square matrix consisting of M×M elements with element spacing of Nd, where $d\leq \frac{\lambda }{2}$, λ are signal wavelengths. *M* and *N* are coprime, and M<N. As shown in Figure 1, it is a coprime array with M=3 and N=4.

Assume that K sources are incident on the array from $\left(\theta_{k},\phi_{k}\right),k=1,2,\ldots ,K$ and $\theta_{k}$ and $\phi_{k}$ represent the elevation and azimuth angles of the k-th source, respectively. The direction matrix of the first subarray can be expressed as follows:

(1) $$A_{1}=A_{1y}⊙A_{1x}$$

where $A_{1x}$ represents the directional matrix of subarray 1 in the *x*-axis direction and $A_{1y}$ represents the directional matrix of subarray 1 in the *y*-axis direction.

(2) $$A_{1x}=\left[\begin{array}{ccc} \begin{array}{cc} 1 & 1\\ e^{j2\pi Nd\cos\phi_{1}\sin\theta_{1}/\lambda } & e^{j2\pi Nd\cos\phi_{2}\sin\theta_{2}/\lambda } \end{array} & \cdots & \begin{array}{c} 1\\ e^{j2\pi Nd\cos\phi_{K}\sin\theta_{K}/\lambda } \end{array}\\ \vdots & \ddots & \vdots \\ \begin{array}{cc} e^{j2\pi \left(M-1\right)Nd\cos\phi_{1}sin\theta_{1}/\lambda } & e^{j2\pi \left(M-1\right)Nd\cos\phi_{2}\sin\theta_{2}/\lambda } \end{array} & \cdots & e^{j2\pi \left(M-1\right)Nd\cos\phi_{K}\sin\theta_{K}/\lambda } \end{array}\right].$$

(3) $$A_{1y}=\left[\begin{array}{ccc} \begin{array}{cc} 1 & 1\\ e^{j2\pi Nd\sin\phi_{1}\sin\theta_{1}/\lambda } & e^{j2\pi Nd\sin\phi_{2}\sin\theta_{2}/\lambda } \end{array} & \cdots & \begin{array}{c} 1\\ e^{j2\pi Nd\sin\phi_{K}\sin\theta_{K}/\lambda } \end{array}\\ \vdots & \ddots & \vdots \\ \begin{array}{cc} e^{j2\pi \left(M-1\right)Nd\sin\phi_{1}sin\theta_{1}/\lambda } & e^{j2\pi \left(M-1\right)Nd\sin\phi_{2}\sin\theta_{2}/\lambda } \end{array} & \cdots & e^{j2\pi \left(M-1\right)Nd\sin\phi_{K}\sin\theta_{K}/\lambda } \end{array}\right].$$

These equations define the direction matrices for subarray 1 in the *x* and *y* directions. Matrices $A_{1x}$ and $A_{1y}$ capture the phase shifts due to the spatial separation of array elements, which are crucial for DOA estimation. Similarly, the direction matrix and directional matrices in the *x*-axis and *y*-axis directions of subarray 2 are expressed as follows:

(4) $$A_{2}=A_{2y}⊙A_{2x}.$$

(5) $$A_{2x}=\left[\begin{array}{ccc} \begin{array}{cc} 1 & 1\\ e^{j2\pi Md\cos\phi_{1}\sin\theta_{1}/\lambda } & e^{j2\pi Md\sin\phi_{2}\cos\theta_{2}/\lambda } \end{array} & \cdots & \begin{array}{c} 1\\ e^{j2\pi Md\sin\phi_{K}\cos\theta_{K}/\lambda } \end{array}\\ \vdots & \ddots & \vdots \\ \begin{array}{cc} e^{j2\pi \left(N-1\right)Md\cos\phi_{1}sin\theta_{1}/\lambda } & e^{j2\pi \left(N-1\right)Md\cos\phi_{2}\sin\theta_{2}/\lambda } \end{array} & \cdots & e^{j2\pi \left(N-1\right)Md\cos\phi_{K}\sin\theta_{K}/\lambda } \end{array}\right]$$

(6) $$A_{2y}=\left[\begin{array}{ccc} \begin{array}{cc} 1 & 1\\ e^{j2\pi Md\sin\phi_{1}\sin\theta_{1}/\lambda } & e^{j2\pi Md\sin\phi_{2}\sin\theta_{2}/\lambda } \end{array} & \cdots & \begin{array}{c} 1\\ e^{j2\pi Md\sin\phi_{K}\sin\theta_{K}/\lambda } \end{array}\\ \vdots & \ddots & \vdots \\ \begin{array}{cc} e^{j2\pi \left(N-1\right)Md\sin\phi_{1}sin\theta_{1}/\lambda } & e^{j2\pi \left(N-1\right)Md\sin\phi_{2}\sin\theta_{2}/\lambda } \end{array} & \cdots & e^{j2\pi \left(N-1\right)Md\sin\phi_{K}\sin\theta_{K}/\lambda } \end{array}\right]$$

Therefore, the direction matrix (A) of the coprime array is expressed as follows:

(7) $$A=\left[\begin{array}{c} A_{1}\\ A_{2} \end{array}\right]$$

The received signal of the array is expressed as follows:

(8) $$x\left(t\right)=As\left(t\right)+n\left(t\right)$$

where A represents the direction matrix of the array, $n\left(t\right)$ is additive Gaussian white noise, and $s\left(t\right)$ is the source vector. This equation models the received signal at the array, where A is the direction matrix, s(t) is the source signal vector, and n(t) is the additive noise. This model is fundamental for DOA estimation, as it relates the received signals to the source directions.

The received signal of a sparse array needs to be virtualized to obtain sufficient spatial resolution before it can be used for two-dimensional DOA estimation.

According to [29], the covariance matrix of the received signal (x(t)) can be expressed as follows:

(9) $$R=E\left[x\left(t\right)x^{H}\left(t\right)\right]=AR_{s}A^{H}+\sigma_{n}^{2}I_{N}$$

where $I\in C^{N\times N}$ is an identity matrix, $R_{s}=\mathrm{diag}\left(\sigma_{1}^{2},\sigma_{2}^{2},\ldots ,\sigma_{K}^{2}\right)$, $\sigma_{k}^{2}$ represents the average power of the *k*-th signal, and k=1,2,…,K. The covariance matrix (R) of the received signals is used to extract information about the signal and noise subspaces. This matrix is essential for subspace-based DOA estimation methods like MUSIC.

When the number of snapshots is L, the covariance matrix of the received signal can be calculated as follows:

(10) $$R_{xx}=\frac{1}{L}\sum_{t=1}^{L}x\left(t\right)x^{H}\left(t\right)$$

Continuing to vectorize the covariance matrix, according to reference [30], the autocorrelation matrix of the received signal can be vectorized as follows:

(11) $$\tilde{z}=vec\left(R_{xx}\right)=\mathrm{Bp}+\sigma_{n}^{2}I_{n}.$$

where $B=A^{*}⊙A$ is the direction matrix of the virtual array, $p={\left[s_{1}^{2},s_{2}^{2},\ldots ,s_{K}^{2}\right]}^{T}$, $\sigma_{n}^{2}$ is the noise power, $s_{K}^{2}$ is the power of the k-th signal, and $I_{n}=vec\left(I\right)$. Then, $\tilde{z}$ is sorted by phase, and redundancy is removed to obtain vector z, which is the received signal of the virtual array [31] of the sparse array. Vectorizing the covariance matrix transforms it into a form suitable for constructing the virtual array. Matrix B represents the direction matrix of the virtual array, which is crucial for increasing the degrees of freedom (DOFs) and improving DOA estimation.

To determine the positions of the elements in a virtual array, the concepts of the sum co-array and difference co-array [32,33] are required. The definitions of common a matrix and differential matrix are presented as follows:

**Definition** **1** ([6])**.** *Consider a linear sparse array of N elements with element positions of $S=\left[s_{1},s_{2},\ldots ,s_{N}\right]d$, where the element positions of the difference co-array are represented as follows:*

(12) $$D_{c}=\left\{\left(s_{i}-s_{j}\right)d\;i,j=1,2,\ldots ,N\right\}$$

**Definition** **2** ([6])**.** *The position numbers of the summation elements of the sparse array mentioned in Definition 1 are represented as follows:*

(13) $$D_{s}=\left\{\left(s_{i}+s_{j}\right)d\;i,j=1,2,\ldots ,N\right\}$$

The direction vector of a virtual planar array of commensurate planar arrays can be regarded as the Kronecker product of the direction vectors of the differential arrays located on the *x*-axis and *y*-axis, so the direction vector of the virtual planar array can be regarded as the sum of the direction vectors of the two differential arrays. Therefore, the virtual planar array can be represented as the sum-difference co-array (SDCA) of commensurate planar arrays.

The location set of the virtual array represented by the differential joint array (DCA) of the sparse surface array is defined as follows:

(14) $$D=\left\{\left.n_{1}-n_{2}\right|n_{1},n_{2}\in T\right\}$$

where T represents the set of physical array positions of a sparse array.

### 2.2. Holes in Traditional Coprime Planar Arrays

The set of physical element positions for traditional coprime planar arrays is expressed as follows:

$$L_{CPA}=L_{1}\cup L_{2}$$

$$L_{1}=\left\{\left.\left(p_{1}M,p_{2}M\right)\right|0\leq p_{1},p_{2}\leq N-1\right\}$$

(15) $$L_{2}=\left\{\left.\left(q_{1}N,q_{2}N\right)\right|0\leq q_{1},q_{2}\leq M-1\right\}$$

where M<N, *M*, and *N* are coprime.

According to Equation (14), the DCA of a CPA is expressed as follows:

(16) $$D_{CPA}=\left\{n_{1}-n_{2}\left|n_{1},n_{2}\in L_{CPA}\right.\right\}$$

**Figure 2:**
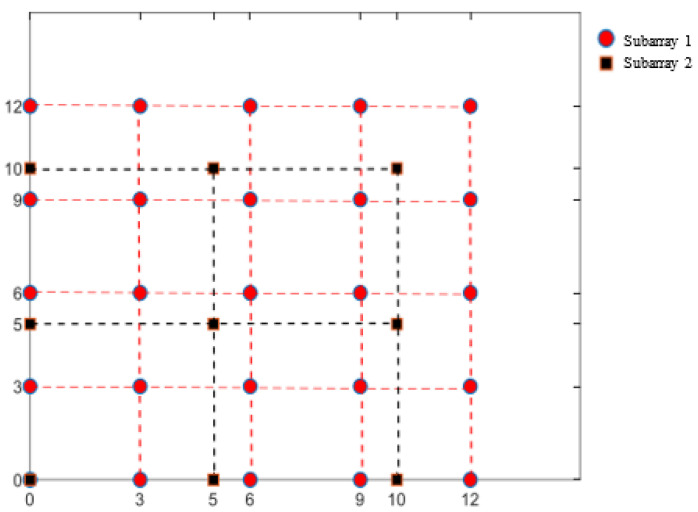
Physical array of a coprime planar array (M = 3, N = 5).

**Figure 3:**
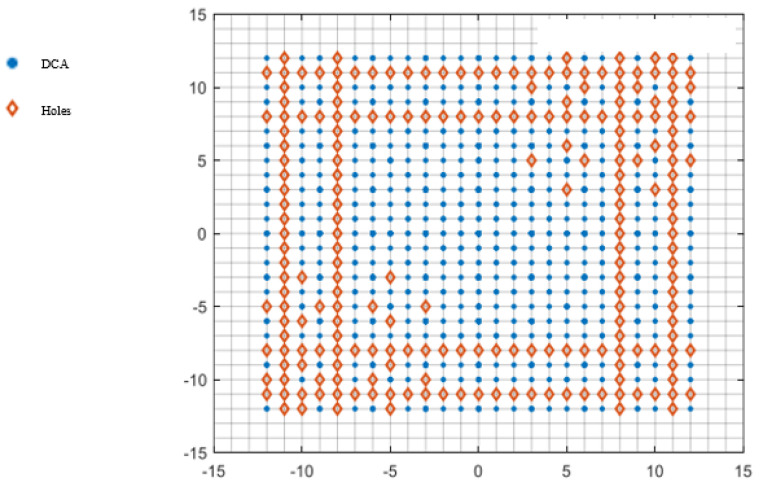
Virtual array of a CPA.

Figure 2 shows a CPA with M = 3 and N = 5; its DCA is shown in Figure 3. The continuous part of the DCA is $\left\{(x,y)|-(N-1)\leq x,y\leq N-1\right\}$.

Due to the symmetrical distribution of the DCA, we only need to consider the holes in the first and second quadrants, which are expressed as follows:

$$H=H_{1}\cup H_{2}$$

(17) $$H_{1}=H_{11}\cup H_{12}\cup H_{13}\cup H_{14}$$

where

$$H_{11}=\left\{\left(x,y\right)\left|x=aM+bN,a\geq 1,b\geq 1,0\leq x,y\leq \left(N-1\right)M\right.\right\}$$

$$H_{12}=\left\{\left(x,y\right)\left|x=aN,y=bM,a\geq 1,b\geq 1,0\leq x,y\leq \left(N-1\right)M\right.\right\}$$

$$H_{13}=\left\{\left(x,y\right)\left|x=aM,y=bN,a\geq 1,b\geq 1,0\leq x,y\leq \left(N-1\right)M\right.\right\}$$

(18) $$H_{14}=\left\{\left(x,y\right)\left|y=aM+bN,a\geq 1,b\geq 1,0\leq x,y\leq \left(N-1\right)M\right.\right\}$$

and

$$H_{11}=\left\{\left(x,y\right)\left|x=aM+bN,a\leq -1,b\leq -1,-\left(N-1\right)M\leq x\leq 0,0\leq y\leq \left(N-1\right)M\right.\right\}$$

(19) $$H_{12}=\left\{\left(x,y\right)\left|x=aM+bM,a\geq 1,b\geq 1,-\left(N-1\right)M\leq x\leq 0,0\leq y\leq \left(N-1\right)M\right.\right\}$$

Equations (17)–(19) define the locations of holes in different quadrants of the traditional coprime planar array. These holes impact the continuity of the virtual array, which is crucial for improving DOFs.

### 2.3. Supplementary Coprime Planar Array

There are some key holes in the first and third quadrants, which are sparsely located in the range of −(M+N) to (M+N), disrupting the continuity of the DCA in this range. For these key holes belonging to $H_{12}$ and $H_{13}$ in the first quadrant, a supplementary method can be proposed by adding a small number of elements to fill these holes.

Consider the element of hole $H_{12}$ in Equation (18), assuming M<N, the key hole to be filled is $H_{12}^{\prime }=\left\{\left(N,iM\right)\left|1\leq i\leq I,I=\left[\frac{N}{M}+1\right]\right.\right\}$, where $\left[\cdot \right]$ represents the maximum integer that does not exceed this number. The array elements (N,KM) are supplemented to fill the key hole ($H_{12}^{\prime }$).

Then, a set of elements ($\left(0,kM\right),0\leq k\leq I-1$) is selected because $0\leq k\leq I-1=\left[\frac{N}{M}\right]<\frac{N}{M}<N-1$, and all elements at $\left(0,kM\right),0\leq k\leq I-1$ are located in the original CPA.

The difference between the supplementary element (N,iM) and the original CPA element ($\left(0,kM\right),0\leq k\leq I-1$) can be expressed as $C=\left\{\left(N,gM\right)\left|g=I-k\right.\right\}=H_{12}^{\prime }$.

Similarly, the key hole that needs to be filled for the element of hole $H_{13}$ is $H_{13}^{\prime }=\left\{\left(iM,N\right)\left|1\leq i\leq I,I=\left[\frac{N}{M}+1\right]\right.\right\}$, and the position of the added element is (IM,N). This type of array, which adds two additional elements ((N,IM) and (IM,N)) to the traditional CPA, is a complementary coprime planar array (CCPA), where $I=\left[\frac{N}{M}+1\right]$. The element positions of the CCPA can be represented as follows:

(20) $$Q_{CCPA}=L_{CPA}\cup \left\{\left(N,IM\right),\left(IM,N\right)\right\}$$

The DCA of the CCPA is expressed as:

(21) $$D_{CCPA}=\left\{n_{1}-n_{2}\left|n_{1},n_{2}\in Q_{CCPA}\right.\right\}=D_{C}\cup D_{O}=\left\{\left(v_{x},v_{y}\right)\left|-M-N+1<v_{x},v_{y}<M+N+1\right.\right\}\cup D_{O}$$

where $D_{C}$ and $D_{O}$ refer to the continuous and peripheral parts of the DCA, respectively.

**Figure 4:**
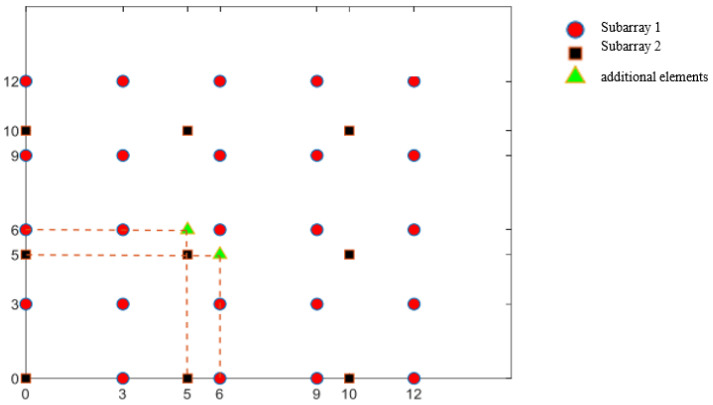
Physical array of a complementary coprime planar array (M = 3, N = 5).

**Figure 5:**
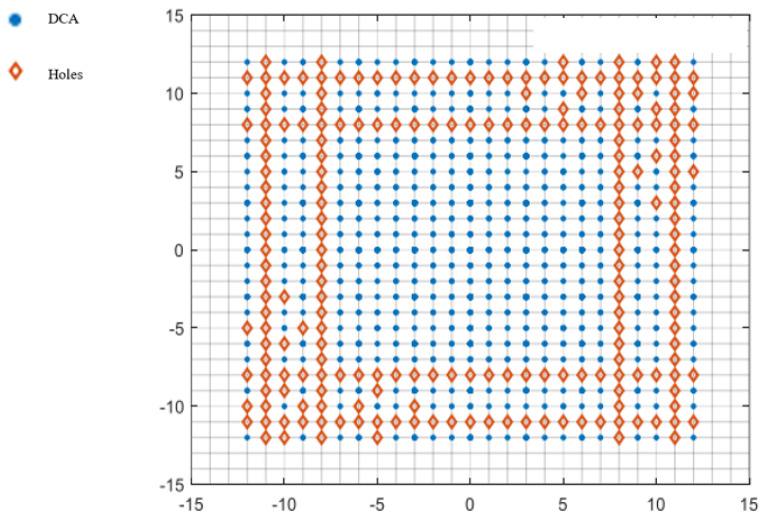
Virtual array of a CCPA.

The CPA in Figure 2 only needs two additional elements to be added at (5,6) and (6,5), as shown in Figure 4, to fill all the holes in its DCA in $\left\{\left(x,y\right)\left|-8<\right.x,y<8\right\}$. The DCA of such a CCPA is shown in Figure 5.

## 3. Two-Dimensional DOA Estimation

This section introduces the dimensionality-reduction root MUSIC algorithm, transforming 2D spectral search into two 1D polynomial root-finding problems, significantly reducing computational complexity.

### 3.1. Spatial Smoothing Process

The spatial smoothing of a uniform array, formed after the virtualization of a sparse array, is achieved through a systematic process. First, the entire array is divided into several identical and overlapping subarrays. Then, the covariance matrix of each subarray is calculated based on the single-snapshot data it receives. Finally, the original covariance matrix is replaced with the average of the covariance matrices of all subarrays.

It is particularly crucial in addressing the rank deficiency problem encountered in one-dimensional DOA estimation. In this scenario, the virtualization of the sparse array often results in data equivalent to a single-snapshot signal. Single-snapshot signals lead to a loss of rank in the covariance matrix, which in turn, undermines the effectiveness of high-resolution subspace-based algorithms such as MUSIC. As a result, the spatial smoothing method is employed in this paper to decorrelate the data, thereby restoring the rank of the covariance matrix and enabling the effective application of subspace algorithms.

By adopting spatial smoothing for the decorrelation preprocessing of the virtualized sparse array data, the proposed approach ensures reliable DOA estimation, even in challenging single-snapshot scenarios. This preprocessing step lays the foundation for subsequent high-resolution direction-finding techniques.

**Figure 6:**
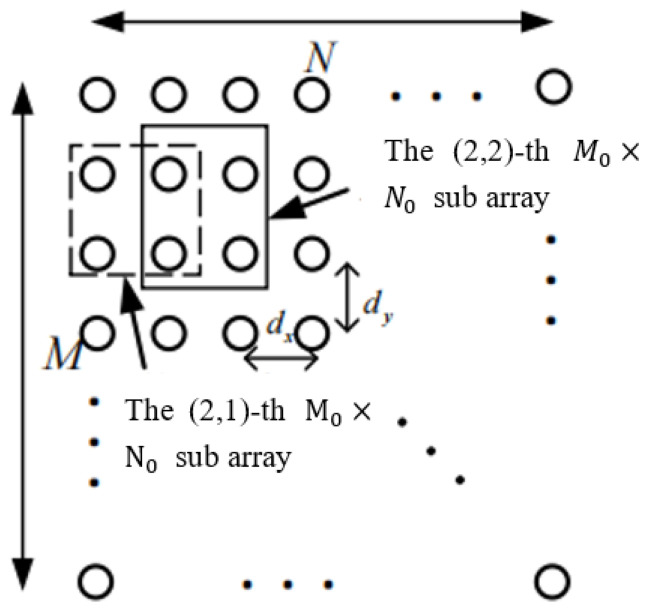
Schematic diagram of two-dimensional space smoothing.

The subarray is a uniform surface array of $M_{0}\times N_{0}$, stacked as shown in the Figure 6, with a total of $M_{s}=\left(M-M_{0}+1\right)$ subarrays in the *x*-axis direction and $N_{s}=\left(N-N_{0}+1\right)$ subarrays in the *y*-axis direction, for a total of $M_{s}\times N_{s}$ subarrays. Therefore, taking the (1,1)-th subarray as the reference array, the element positions of the (m,n)-th subarray are given by the following set:

(22) $$S_{m,n}=\left\{\left(x,y\right)\left|m\leq x\leq m+M_{0}-1,n\leq y\leq n+N_{0}-1\right.\right\}$$

For the (m,n)-th subarray of the CCPA, the received signal is denoted as $z_{mn}$, resulting in a data covariance matrix expressed as follows:

(23) $$R_{mn}=z_{mn}z_{mn}^{H}$$

The covariance matrix of the received signals is calculated from all subarrays in sequence, taking the average value. The final covariance matrix after spatial smoothing is represented as follows:

(24) $$R=\frac{1}{M_{s}\times N_{s}}\sum_{m=1}^{M_{s}}\sum_{n=1}^{N_{s}}R_{mn}$$

Due to the discontinuities in the virtual array of sparse arrays, the covariance matrix becomes rank-deficient. By applying the aforementioned spatial smoothing algorithm, the correlation among coherent signals [34] can be disrupted, thereby restoring the full rank of the covariance matrix. Spatial smoothing is applied to decorrelate coherent signals and restore the rank of the covariance matrix. This step is necessary for effective DOA estimation using subspace-based methods.

### 3.2. Dimensionality-Reduction Root MUSIC Algorithm

According to the array signal processing theory, the covariance matrix (R) can be decomposed into two parts: the noise subspace and signal subspace, expressed as follows:

(25) $$R=E_{s}\Sigma_{s}E_{s}^{H}+E_{n}\Sigma_{n}E_{n}^{H}$$

where $\Sigma_{s}=\mathrm{diag}\left\{\lambda_{1},\lambda_{2},\cdots ,\lambda_{K}\right\}\in C^{K\times K}$ is a diagonal matrix composed of K large eigenvalues ($\lambda_{k},k=1,2,\ldots ,K$) and $\Sigma_{n}=\mathrm{diag}\left\{\lambda_{K+1},\lambda_{K+2},\cdots ,\lambda_{M}\right\}\in C^{\left(M-K\right)\times \left(M-K\right)}$ is a diagonal matrix consisting of the remaining (M − K) small eigenvalues. $E_{n}$ and $E_{s}$ are eigenvectors corresponding to smaller eigenvalues and larger eigenvalues, respectively.

According to the orthogonal characteristics of signal vectors and noise subspaces, the two-dimensional MUSIC spectral function is expressed as follows:

(26) $$P_{2D-MUSIC}\left(\theta ,\phi \right)=\frac{1}{a^{H}\left(\theta ,\phi \right)E_{n}E_{n}^{H}a\left(\theta ,\phi \right)}$$

where $a\left(\theta ,\phi \right)=a_{y}\left(\theta ,\phi \right)⊗a_{x}\left(\theta ,\phi \right)\in C^{M_{0}N_{0}\times 1}$, $a_{y}\left(\theta ,\phi \right)=\left[1,e^{j2\pi d\sin\phi \sin\theta /\lambda }\right.$, …, ${\left.e^{j2\pi d\left(M-1\right)\sin\phi \sin\theta /\lambda }\right]}^{T}$, $a_{x}\left(\theta ,\phi \right)={\left[1,e^{j2\pi d\cos\phi \sin\theta /\lambda },\ldots ,e^{j2\pi d\left(N-1\right)\cos\phi \sin\theta /\lambda }\right]}^{T}$, and $E_{n}$ represents the noise subspace of the virtual array. The 2D-MUSIC spectral function is used to estimate the DOA by searching for peaks in the spatial spectrum. The function leverages the orthogonality between the signal and noise subspaces.

We define u=sinϕsinθ, v=cosϕsinθ. Therefore, the direction vector can be expressed as $a_{y}\left(\theta ,\phi \right)=a_{y}\left(u\right)={\left[1,e^{j2\pi du/\lambda },\ldots ,e^{j2\pi d\left(M-1\right)u/\lambda }\right]}^{T}$, $a_{x}\left(\theta ,\phi \right)=a_{x}\left(v\right)={\left[1,e^{j2\pi dv/\lambda },\ldots ,e^{j2\pi d\left(N-1\right)v/\lambda }\right]}^{T}$.

Then, a polynomial is constructed using the denominator in the spectral function as follows:

(27) $$V\left(u,v\right)={\left[a_{y}\left(u\right)⊗a_{x}\left(v\right)\right]}^{H}E_{n}E_{n}^{H}\left[a_{y}\left(u\right)⊗a_{x}\left(v\right)\right]$$

This polynomial is constructed to transform the 2D spectral search into a root-finding problem. By solving for the roots of this polynomial, the DOA estimation problem is simplified. The process of finding the peak of the spectral function through search in the two-dimensional MUSIC algorithm is transformed into a process of finding the values of u and v corresponding to the polynomial expressed as $V\left(u,v\right)=0$.

In order to transform a two-dimensional problem into a one-dimensional solution and achieve separate solutions for u and v, first, the variables for u and v are separated, and the polynomial expressed as $V\left(u,v\right)$ is reconstructed as follows:

(28) $$V\left(u,v\right)=a_{x}^{H}\left(v\right){\left[a_{y}\left(u\right)⊗I_{M}\right]}^{H}E_{n}E_{n}^{H}\left[a_{y}\left(u\right)⊗I_{M}\right]a_{x}\left(v\right)=a_{x}^{H}\left(v\right)Q\left(u\right)a_{x}\left(v\right)$$

and

(29) $$V\left(u,v\right)=a_{y}^{H}\left(u\right){\left[I_{M}⊗a_{x}\left(v\right)\right]}^{H}E_{n}E_{n}^{H}\left[I_{M}⊗a_{x}\left(v\right)\right]a_{y}\left(u\right)=a_{y}^{H}\left(u\right)Q\left(v\right)a_{y}\left(u\right)$$

where

(30) $$Q\left(u\right)={\left[a_{y}\left(u\right)⊗I_{M}\right]}^{H}E_{n}E_{n}^{H}\left[a_{y}\left(u\right)⊗I_{M}\right]$$

(31) $$Q\left(v\right)={\left[I_{N}⊗a_{x}\left(v\right)\right]}^{H}E_{n}E_{n}^{H}\left[I_{N}⊗a_{x}\left(v\right)\right].$$

According to the rank relationship between matrix products, the following conditions must be met:

(32) $$Rank\left(E_{n}E_{n}^{H}\right)\leq Rank\left(E_{n}\right)$$

And because virtual arrays can generally form large-scale uniform arrays, MN≫K; therefore, $Rank\left(E_{n}E_{n}^{H}\right)=MN-K>0$.

Because the noise is non-zero, matrix $E_{n}E_{n}^{H}$ is invertible, which means

(33) $$\det\left\{E_{n}E_{n}^{H}\right\}\neq 0$$

Thus, $\det\left\{Q\left(u\right)\right\}$ is a non-zero polynomial and, obviously, $Q\left(u\right)$ is a factor of the polynomial expressed as $V\left(u,v\right)$ because $Q\left(u\right)$ is only related to the u variable. If the value of u satisfies $\det\left\{Q\left(u\right)\right\}=0$, then it can satisfy $V\left(u,v\right)=a_{x}^{H}\left(v\right)Q\left(u\right)a_{x}\left(v\right)=0$ at the same time. Therefore, the root of $\det\left\{Q\left(u\right)\right\}$ contains the required value of u.

By using a similar method to analyze variable v, the problem of finding the values of u and v among an infinite number of solutions to $V\left(u,v\right)=0$ can be transformed into a problem of finding the roots of two one-dimensional polynomials. Therefore,

(34) $$\det\left\{Q\left(u\right)\right\}=\det\left\{{\left[a_{y}\left(u\right)⊗I_{M}\right]}^{H}E_{n}E_{n}^{H}\left[a_{y}\left(u\right)⊗I_{M}\right]\right\}=0$$

(35) $$\det\left\{Q\left(v\right)\right\}=\det\left\{{\left[I_{N}⊗a_{x}\left(v\right)\right]}^{H}E_{n}E_{n}^{H}\left[I_{N}⊗a_{x}\left(v\right)\right]\right\}=0$$

These equations represent the root-finding process for the polynomial expressed as V(u,v). By solving these equations, the DOA angles (*u* and *v*, respectively) are estimated, significantly reducing the computational complexity compared to traditional 2D spectral search methods. We defining the following:

(36) $$\left\{\begin{array}{c} z_{1}=e^{j2\pi du/\lambda }\\ z_{2}=e^{j2\pi dv/\lambda } \end{array}\right.$$

Hence, the direction vector can be written as follows:

(37) $$a_{y}\left(u\right)={\left[1,e^{j2\pi du/\lambda },\ldots ,e^{j2\pi \left(N-1\right)du/\lambda }\right]}^{T}={\left[1,z_{1},\ldots ,z_{1}^{N-1}\right]}^{T}=a_{y}\left(z_{1}\right)$$

(38) $$a_{x}\left(v\right)={\left[1,e^{j2\pi dv/\lambda },\ldots ,e^{j2\pi \left(N-1\right)dv/\lambda }\right]}^{T}={\left[1,z_{2},\ldots ,z_{2}^{M-1}\right]}^{T}=a_{x}\left(z_{2}\right)$$

To estimate u and v, $\left[a_{y}\left(u\right)⊗I_{M}\right]$ is replaced with $z_{1}^{N-1}{\left[a_{y}^{T}\left(z_{1}^{-1}\right)⊗I_{M}\right]}^{H}$ and ${\left[I_{N}⊗a_{x}\left(v\right)\right]}^{H}$ is replaced with $z_{2}^{M-1}{\left[I_{N}⊗a_{x}^{T}\left(z_{2}^{-1}\right)\right]}^{H}$, which means the following:

(39) $$\det\left\{Q\left(z_{1}\right)\right\}=\det\left\{z_{1}^{N-1}{\left[a_{y}^{T}\left(z_{1}^{-1}\right)⊗I_{M}\right]}^{H}E_{n}E_{n}^{H}\left[a_{y}^{T}\left(z_{1}\right)⊗I_{M}\right]\right\}=0$$

(40) $$\det\left\{Q\left(z_{2}\right)\right\}=\det\left\{z_{2}^{M-1}{\left[I_{N}⊗a_{x}^{T}\left(z_{2}^{-1}\right)\right]}^{H}E_{n}E_{n}^{H}\left[I_{N}⊗a_{x}\left(z_{2}\right)\right]\right\}=0$$

Because the order of $\det\left\{Q\left(z_{1}\right)\right\}$ and $\det\left\{Q\left(z_{2}\right)\right\}$ is even, we can take the K roots (${\hat{z}}_{11},{\hat{z}}_{12},\ldots ,{\hat{z}}_{1K}$) closest to the unit circle in Equation (39) to find ${\hat{u}}_{k}$ and take the K roots (${\hat{z}}_{21},{\hat{z}}_{22},\ldots ,{\hat{z}}_{2K}$) closest to the unit circle in Equation (40) to find ${\hat{v}}_{i}$.

(41) $${\hat{u}}_{k}=angle\left({\hat{z}}_{1k}\right)\lambda /2\pi d,k=1,2,\ldots ,K$$

(42) $${\hat{v}}_{i}=angle\left({\hat{z}}_{2i}\right)\lambda /2\pi d,i=1,2,\ldots ,K$$

These equations provide the theoretical basis for solving the two-dimensional DOA estimation problem efficiently. Reducing the dimensionality of the computational process significantly lowers the computational burden while maintaining estimation accuracy. This approach ensures the practical feasibility of applying RD-root-MUSIC in scenarios with two-dimensional data.

### 3.3. Parameter Pairing and Two-Dimensional DOA Estimation

Since ${\hat{u}}_{k}$ and ${\hat{v}}_{i}$ are estimated separately, they need to be paired to achieve two-dimensional DOA estimation. For this reason, we construct the following function:

(43) $$V_{k,i}=\underset{i=1,2,\ldots ,K}{\arg\min}∥{\left[a_{y}\left({\hat{u}}_{k}\right)⊗a_{x}\left({\hat{v}}_{i}\right)\right]}^{H}E_{n}E_{n}^{H}\left[a_{y}\left({\hat{u}}_{k}\right)⊗a_{x}\left({\hat{v}}_{i}\right)\right]∥$$

where $a_{y}\left({\hat{u}}_{k}\right)$ and $a_{x}\left({\hat{v}}_{i}\right)$ represent the direction vectors reconstructed by ${\hat{u}}_{k}$ and ${\hat{v}}_{i}$, respectively.

Obviously, a total of $K^{2}V_{k,i}$ values can be calculated. For each k, we can obtain the minimum ($V_{k,i}\left(1\leq i\leq K\right)$) and denote the corresponding i as $i^{\prime }$. Then, we consider ${\hat{u}}_{k}$ and ${\hat{v}}_{i}$ to be paired.

Therefore, the result of two-dimensional DOA estimation is expressed as follows:

(44) $${\hat{\theta }}_{k}=\sin^{-1}\left(\sqrt{{\hat{u}}_{k}^{2}+{\hat{v}}_{i^{\prime }}^{2}}\right),1\leq k\leq K$$

(45) $${\hat{\phi }}_{k}=\tan^{-1}\left(\frac{{\hat{u}}_{k}}{{\hat{v}}_{i^{\prime }}}\right),1\leq k\leq K$$

These equations provide the final estimates of the elevation (θ) and azimuth (ϕ) angles. The pairing of ${\hat{u}}_{k}$ and ${\hat{v}}_{i}$ ensures consistent and accurate DOA estimation. The final expression pairs the two-dimensional DOA angles, enabling precise estimation of the target directions. This method leverages the parameter-pairing strategy to ensure that the estimated azimuth and elevation angles are consistent, improving the reliability and accuracy of the algorithm in practical applications.

### 3.4. Main Steps of the Algorithm

The main steps of the dimensionality-reduction root MUSIC algorithm proposed in this section can be summarized as follows:

First, the covariance matrix ($R_{xx}$) of the received signal (x(t)) is calculated using Equation (10); then, the received signal (z) of the difference joint array is obtained using Equation (11).

Next, the middle corresponding part of the received signal z is extracted based on the position of the continuous part of the differential joint array, and the received signal ($z_{1}$) of the differential joint array is obtained.

Then, the spatially smoothed covariance matrix (R) is obtained using Equations (23) and (24), and eigenvalue decomposition is performed to obtain the noise subspace ($E_{n}$).

After that, polynomial V(u,v) is constructed according to Equation (27); then, the dimensionality of V(u,v) is reduced to obtain Equations (34) and (35).

Subsequently, ${\hat{u}}_{k}$ and ${\hat{v}}_{i}$ are calculated based on Equations (41) and (42), respectively.

Finally, ${\hat{u}}_{k}$ and ${\hat{v}}_{i}$ are paired, and the two-dimensional DOA estimation results are calculated based on Equations (44) and (45).

## 4. Performance Analysis

This section evaluates the performance of the proposed complementary coprime planar array (CCPA) and the dimensionality-reduction root MUSIC algorithm through various simulations and analyses.

### 4.1. Continuous Degrees-of-Freedom Analysis of Arrays

The proposed CCPA achieves a continuous range of the virtualized DCA as $\left(2U_{x}+1\right)\times \left(2U_{y}+1\right)$ after virtualization [35], where $U_{x}=M+N-1$ and $U_{y}=M+N-1$. For the UPA, the continuous range of the virtualized DCA is $\left(2T_{x}-1\right)\times \left(2T_{y}-1\right)$. For the CPA, the continuous range of the virtualized DCA is ${\left(2N_{\mathrm{CPA}}-1\right)}^{2}$. Table 1 compares the continuous DOFs achievable by three different arrays. It can be seen that the DCA of the CCPA structure proposed in this paper has relatively high continuous DOFs.

### 4.2. Analysis of Algorithm Advantages

The advantages of the algorithm proposed in this section are outlined as follows:

Firstly, this algorithm converts two-dimensional polynomials into one-dimensional polynomials without the need for spectral search, significantly reducing computational complexity.

Secondly, compared to 2D-MUSIC and dimensionality-reduction MUSIC algorithms, this algorithm offers substantially lower computational complexity while maintaining competitive performance.

Finally, the 2D-DOA estimation accuracy of this algorithm surpasses that of the 2D-PM and 2D-ESPRIT algorithms and closely approaches the accuracy levels of 2D-MUSIC and dimensionality-reduction MUSIC algorithms.

### 4.3. Analysis of Algorithm Complexity

In this section, the dimensionality-reduction root MUSIC algorithm is used to estimate the two-dimensional DOA results for the CCPA. Assuming that the CCPA is set to (M, N), the continuous part of its DCA is (2U−1)×(2U−1), where U=M+N. The complexity of calculating the covariance matrix using this method is $O(U^{4})$. $\bar{R}$ features with a complexity of $O(U^{6})$ are decomposed. The complexity of finding the roots of a quadratic polynomial is $O(4U{\left(U-1\right)}^{3})$, and the computational cost of the final pairing process is $O(K^{2}\left(U^{2}+1\right)\left(U^{2}-K\right))$; thus, the computational complexity of the dimensionality-reduction root-finding MUSIC algorithm is $O(U^{4}+U^{6}+4U{\left(U-1\right)}^{3}+K^{2}\left(U^{2}+1\right)\left(U^{2}-K\right))$.

**Figure 7:**
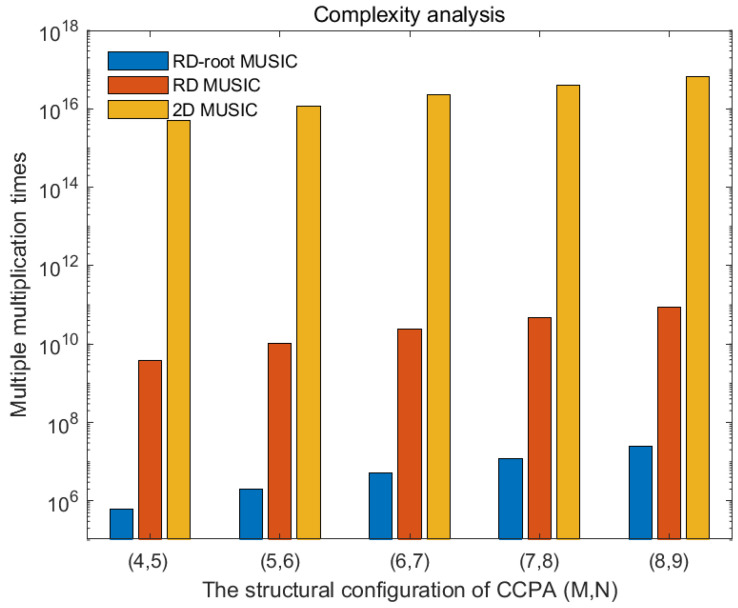
Comparison of complexities of different algorithms.

Table 2 provides a detailed comparison of the computational complexities among the proposed dimensionality-reduction root MUSIC algorithm, the two-dimensional MUSIC algorithm, and the dimensionality-reduction MUSIC algorithm. As shown in the table, the proposed method achieves a lower overall complexity than the dimensionality-reduction MUSIC algorithm and significantly outperforms the two-dimensional MUSIC algorithm in terms of computational efficiency. These results underscore the advantage of the proposed approach in simplifying operations while maintaining effectiveness. Figure 7 depicts the number of multiplication operations required by the three algorithms under varying conditions. The data clearly demonstrate that the proposed dimensionality-reduction root MUSIC algorithm involves fewer multiplications compared to the dimensionality-reduction MUSIC algorithm, with an even more pronounced reduction relative to the two-dimensional MUSIC algorithm. This visualization effectively highlights the computational benefits of the proposed algorithm, particularly in scenarios with higher complexity demands.

### 4.4. Comparison of RMSE for Different Arrays

In this section, RD-root-MUSIC is exploited to estimate 2D-DOA. A CPA with (M = 2, N = 7) and a UPA with ($T_{x}=6,T_{y}=9$) are used for comparison, with totals of 52 and 54 sensors, respectively. The proposed CCPA is designed with M = 4 and N = 5; therefore a total of 42 sensors are used. The simulations compare the RMSE performance of different arrays and demonstrate the superiority of the proposed method.

**Figure 8:**
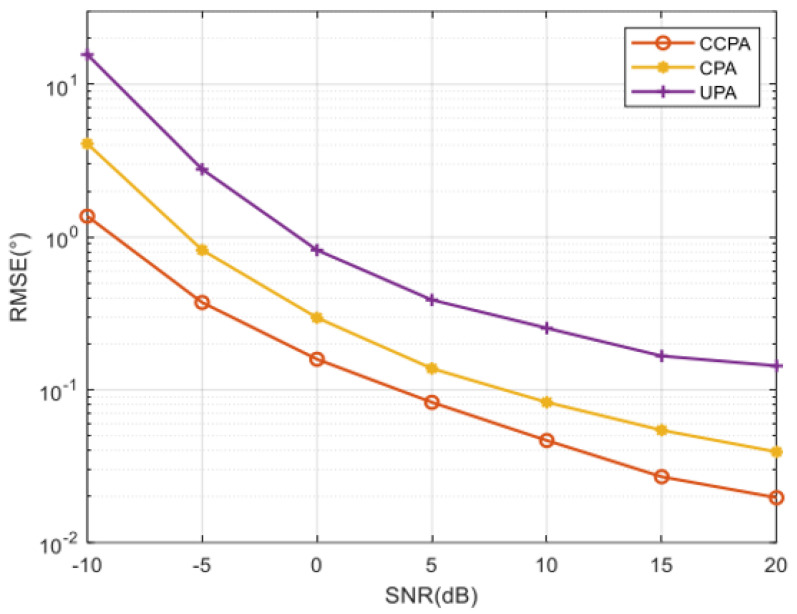
RMSE comparison with SNR variation for different arrays.

The first simulation, illustrated in Figure 8, compares the RMSEperformance of the UPA, CPA, and the proposed CCPA with varying SNR values while maintaining a fixed snapshot number of J=200. As shown in Figure 8, as the signal-to-noise ratio increases, all arrays achieve better estimation results, with their root mean square errors RMSEs) decreasing rapidly. Among them, the proposed CCPA achieves the lowest RMSE, demonstrating superior accuracy compared to its competitors. In addition, within a wider range of signal-to-noise ratios, the CCPA structure achieves a smaller RMSE than other arrays. This indicates that under the same conditions, the two-dimensional DOA estimation performance of the CCPA structure is superior to that of the UPA and CPA structures.

**Figure 9:**
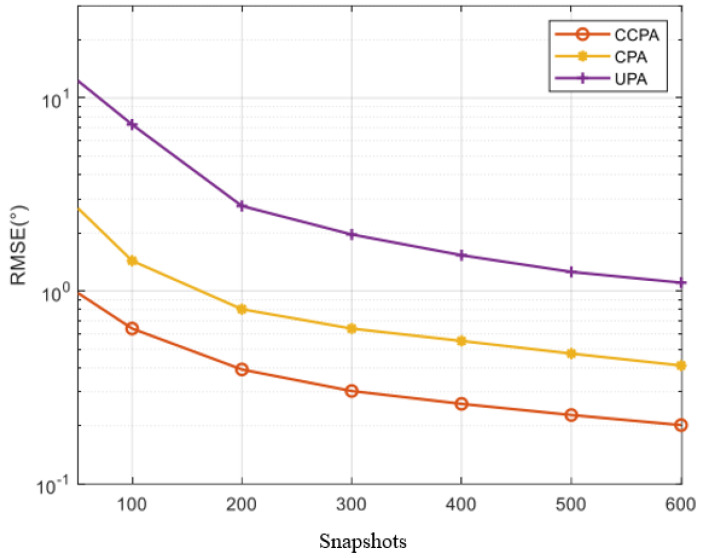
RMSE comparison with snapshot variation for different arrays.

Figure 9 shows the RMSE comparison of the CCPA, UPA, and CPA with changes in the number of snapshots, as shown in Figure 9, while maintaining a signal-to-noise ratio of SNR = −5 dB. As the number of snapshots increases, all arrays obtain better estimation results, and all root mean square errors decrease rapidly. Similar to the results shown in Figure 8, within a wider range of snapshot counts, the CCPA exhibits better performance than other arrays.

### 4.5. The Effectiveness of Two-Dimensional DOA Estimation

**Figure 10:**
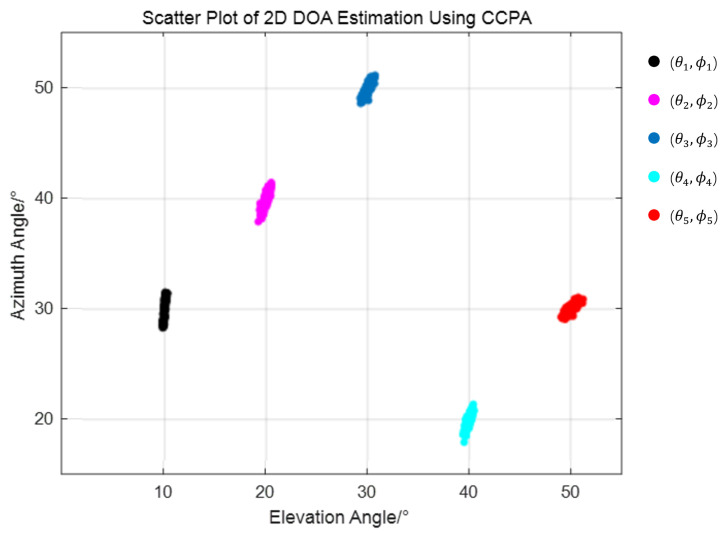
Two-dimensional DOA estimation under a CCPA array.

In order to test the effectiveness of the array and algorithm proposed in this paper, the dimensionality-reduction root MUSIC algorithm was used to estimate the two-dimensional DOA results for the CCPA. The CCPA parameters in the simulation are set to (M = 4, N = 5). Assuming that K = 5 sources are incident on the array from $\left(\theta_{1},\phi_{1}\right)=\left(10,30\right),\left(\theta_{2},\phi_{2}\right)=\left(20,40\right),\left(\theta_{3},\phi_{3}\right)=\left(30,50\right),\left(\theta_{4},\phi_{4}\right)=\left(40,20\right),\left(\theta_{5},\phi_{5}\right)=\left(50,30\right)$, respectively, SNR = 10 dB, the snapshot count is 200, and the experiment is repeated 500 times. Figure 10 verifies the efficiency of the proposed method for 2D-DOA estimation in terms of Scatter figures. As shown in Figure 10, the two-dimensional DOA estimation results of the array are concentrated near the set source direction, and the correct results can be estimated.

### 4.6. The Influence of the Number of Array Elements on the Proposed Algorithm

**Figure 11:**
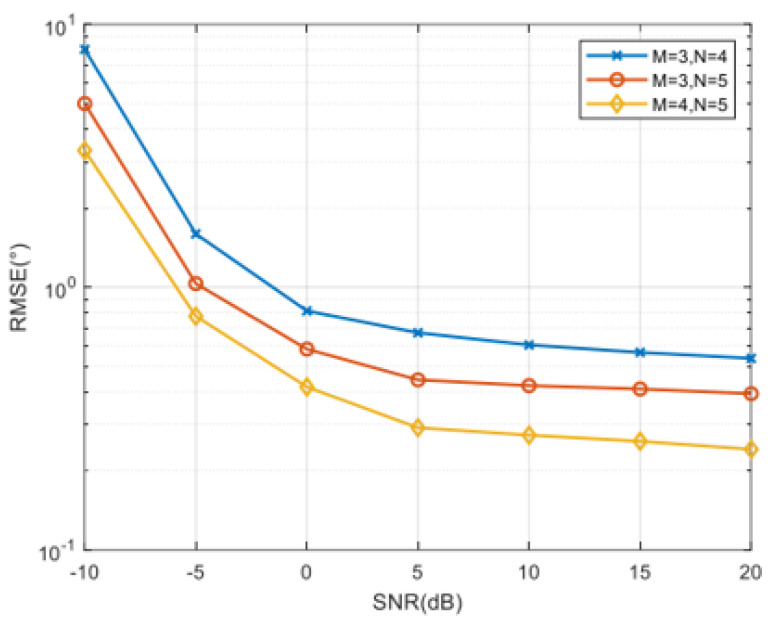
Comparison of RMSE with SNR variation under different numbers of array elements (dimensionality-reduction root MUSIC algorithm).

Assuming that there are K = 3 independent sources incident on three CCPAs with parameters of (M = 3, N = 4), (M = 3, N = 5), and (M = 4, N = 5) from angles of $\left(\theta_{1},\phi_{1}\right)=\left(10,20\right),\left(\theta_{2},\phi_{2}\right)=\left(30,40\right),\left(\theta_{3},\phi_{3}\right)=\left(50,60\right)$, respectively, the algorithm proposed in this paper is used to estimate the incident angles and calculate the RMSE variation with SNR for different numbers of elements. Figure 11 investigates the performance of the proposed method with different structural parameters, revealing that with the increase in the number of array elements, the two-dimensional DOA estimation performance of the algorithm has also improved.

### 4.7. Comparison of RMSE for Different Algorithms

Assuming that K = 2 independent sources are incident on a CCPA with parameters of (M = 2, N = 3) from angles of $\left(\theta_{1},\phi_{1}\right)=\left(20,30\right),\left(\theta_{2},\phi_{2}\right)=\left(40,50\right)$, respectively, the following figures show the simulation and comparison results of the RMSE of the proposed dimensionality-reduction root MUSIC algorithm, dimensionality-reduction MUSIC algorithm, spatially smoothed two-dimensional ESPRIT algorithm, and spatially smoothed two-dimensional PM investigated in this paper.

**Figure 12:**
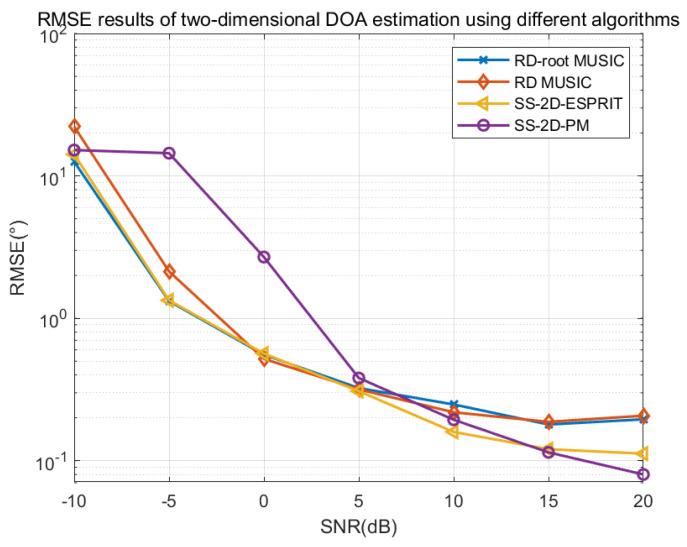
RMSE varies with SNR under different algorithms.

From Figure 12, it can be seen that while maintaining a snapshot count of 200, all algorithms achieve better estimation results with an increase in signal-to-noise ratio. In addition, within a wider range of signal-to-noise ratios, the RMSE error of the dimensionality-reduction root MUSIC algorithm proposed in this section is lower than that of the spatial smoothing two-dimensional ESPRIT algorithm and the spatial smoothing two-dimensional PM and slightly higher than the dimensionality-reduction MUSIC algorithm. However, the dimensionality-reduction root MUSIC algorithm does not require search and can achieve much lower complexity than the dimensionality-reduction MUSIC algorithm.

**Figure 13:**
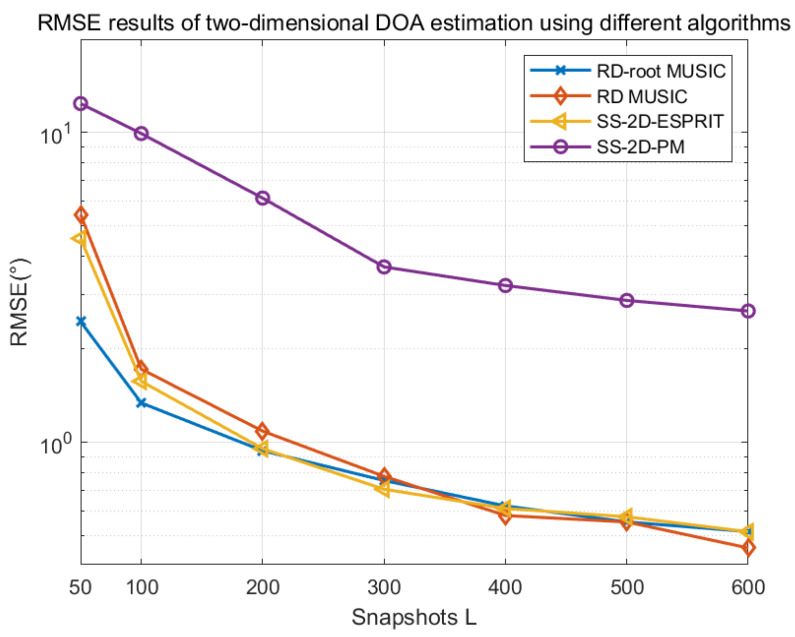
RMSE varies with snapshots under different algorithms.

Figure 13 illustrates the variation of RMSE with the number of snapshots for the dimensionality-reduction root MUSIC algorithm, dimensionality-reduction MUSIC algorithm, and spatially smoothed two-dimensional ESPRIT algorithm while maintaining a signal-to-noise ratio of 0 dB. When the number of snapshots changes from 50 to 600, all algorithms achieve a lower RMSE, indicating that increasing the number of snapshots can improve the DOA estimation performance of the algorithms. In addition, under the simulation conditions, the relationship between the advantages and disadvantages of each algorithm’s estimation performance is consistent with that in Figure 12.

## 5. Conclusions

This paper addresses the challenge of the lack of DOFs of coprime planar arrays and the high complexity of traditional two-dimensional direction-of-arrival estimation methods by introducing an innovative array structure and an efficient algorithm. The CCPA is meticulously designed to achieve higher continuous DOFs in the difference co-array by strategically filling critical structural gaps. Leveraging the extended continuous virtual array provided by the CCPA, the 2D DOAs are estimated using the RD-root-MUSIC algorithm, which effectively transforms the 2D search problem into multiple one-dimensional searches, thereby significantly reducing computational complexity. Simulation and experimental results substantiate the superiority of the proposed array and algorithm in terms of computational efficiency, estimation accuracy, and overall complexity. Notably, the proposed method achieves substantially lower computational demands compared to existing methods while maintaining accuracy comparable to that of the 2D-MUSIC algorithm.

## Figures and Tables

**Table 1:** Continuous degrees of freedom for different arrays.

Array	Number of Array Elements	Continuous DOFs
CCPA	T = 290, M = 8, N = 15	45×45
CPA	$T=288,M_{CPA}=8,N_{CPA}=15$	29×29
UPA	$T=289,T_{x}=17,T_{y}=17$	33×33

**Table 2:** Multiplications for different algorithm.

Algorithm	Multiplications
Dimensionality-reduction root MUSIC algorithm	$O(U^{4}+U^{6}+4U{\left(U-1\right)}^{3}+K^{2}\left(U^{2}+1\right)\left(U^{2}-K\right))$
2D-MUSIC algorithm	$O\left\{U^{4}+U^{6}+n_{1}^{2}\left[\left(U^{2}+1\right)\left(U^{2}-K\right)\right]\right\}$
Dimensionality-reduction MUSIC algorithm	$O\left\{n_{2}K\left[\left(U^{3}+U^{2}\right)\left(U^{2}-K\right)+U^{2}\right]+U^{4}+U^{6}\right\}$

U = M + N, $n_{1}$ is the global search count, and $n_{2}$ is the local search count.

## Data Availability

Data are contained within the article.
